# Finding Cases of Hepatitis C for Treatment Using Automated Screening in the Emergency Department is Effective, but What Is the Cost?

**DOI:** 10.1155/2022/3449938

**Published:** 2022-10-14

**Authors:** David Stephen Prince, Julia Di Girolamo, Joseph Louis Pipicella, Melissa Bagatella, Tahrima Kayes, Frank Alvaro, Michael Maley, Hong Foo, Paul MacConachie Middleton, Miriam Tania Levy

**Affiliations:** ^1^Liverpool Hospital, Sydney, NSW, Australia; ^2^The Ingham Institute for Applied Medical Research, Sydney, NSW, Australia; ^3^The University of NSW, Sydney, NSW, Australia; ^4^NSW Health Pathology, Liverpool, NSW, Australia; ^5^School of Medicine, Western Sydney University, Sydney, NSW, Australia; ^6^South Western Emergency Research Institute, UNSW, Sydney, NSW, Australia; ^7^Sydney Medical School, The University of Sydney, Sydney, NSW, Australia

## Abstract

Case detection remains a major challenge for hepatitis C virus (HCV) elimination. We have previously published results from a pilot of an emergency department (ED) semiautomated screening program, SEARCH; Screening Emergency Admissions at Risk of Chronic HCV. Several refinements to SEARCH have been developed to streamline and reduce cost. All direct costs of HCV testing until direct-acting antiviral (DAA) therapy initiation were calculated. Cost was assessed in 2018 Australian Dollars. A cost analysis of the initial program and refinements are presented. Sensitivity analysis to understand impact of variation in staff time, laboratory test cost, changes in HCV antibody (Ab) prevalence, RNA positivity percentage, and rate of linkage to care was conducted. Impact of refinements (SEARCH (2)) to cost is presented. The total SEARCH pilot, testing 5000 patients was estimated to cost $110,549.52 (range $92,109.79–$129,581.24) comprising of $68,278.67 for HCV Ab testing, $21,568.99 for follow-up and linkage to care of positive patients and $20,701.86 to prepare HCV RNA positive patients for treatment. Internal program refinements resulted in a 25% cost reduction. Following refinements, the cost of HCV antibody screening was $8.46 per test and the total cost per positive HCV Ab, positive HCV RNA, and per treated patient were $611.77, $2,168.64, and $3,566.11, respectively. Our sensitivity analysis indicates costs per HCV case found are modest so long as HCV Ab prevalence was at least 1%. ED screening is an affordable strategy for HCV case detection and elimination.

## 1. Introduction

Despite curative therapies, chronic hepatitis C virus (HCV) infection remains a global health challenge, in part because reaching and treating those infected requires overcoming significant barriers. The transformative nature of direct acting antiviral (DAA) therapy underpinned the development of World Health Organization's (WHO) 2030 elimination goals of 80% of eligible patients receiving treatment to achieve a 65% decrease in HCV-related mortality and an 80% decrease in new HCV infections [1]. In Australia, DAA therapy is already reducing liver related mortality [2].

Overall, it is estimated that only 44% of those with HCV in Australia are diagnosed, linked to care, and treated [3]. Testing without a systematic program relies on the enthusiasm and knowledge of healthcare providers, the attendance by patients for preventive check-ups, patient willingness to undergo risk-based questioning without feeling stigmatised about their background (Indigenous, overseas born (OB), or injecting drug use (IDU) history), and is limited by patient and healthcare worker time. This results in implementation gaps of the current testing recommendations.

In Australia, high treatment uptake in patients with a history of recent IDU has been reported, but gaps remain in women (aOR 0.78; 95% CI 0.72–0.84), patients of Aboriginal and Torres Straight background (ATSI) (aOR 0.75; 95% CI 0.69–0.81), OB individuals (aOR 0.86; 95% CI 0.78–0.96) and in those diagnosed in outer metropolitan areas (aOR 0.90; 95% CI 0.82–0.98) [4]. It seems likely that implementation of testing is more challenging in some groups and novel methods to access these patients for linkage to care (LTC) and treatment are required.

Unlike in the USA, Australian guidelines continue to advocate risk-based rather than universal testing. Patients born in countries with increased prevalence of HCV are recommended to be tested. High HCV prevalence regions include Egypt, Pakistan, Mediterranean and Eastern Europe, Africa, and Asia [5]. HCV in these populations is often acquired via non-IDU routes such as nonsterile healthcare interventions, occupational exposure, or resulting from cultural practices. Nevertheless, there is no systematic screening program or strategy for such populations. Additionally, unlike people who inject drugs (PWID), who may be reached in settings such as prisons, safe injecting services, or opioid substitution services, OB patients are difficult to access in a single setting.

Cost effectiveness studies have shown that testing in a high-risk groups, such PWID or testing all who enter prisons, is cost effective in terms of both cost/quality-adjusted life year (QALY) and the incremental cost effectiveness ratio (ICER) [6, 7]. The majority of these data are derived from overseas countries, particularly, the UK and USA [6–9]. No Australian data have formally assessed the cost utility of prison-based HCV testing and treatment although this is cheaper than standard community-based HCV treatment [10].

Recent work has shown that in the era of DAA therapy, universal onetime screening for HCV is also cost-effective [11, 12]. There is, however, a lack of Australian data and there is no cost analysis of testing OB patients, though we observe they could be accessed systematically in the emergency department (ED). Until recently, testing in pregnancy was not considered to be cost-effective. However, due to improved treatment efficacy and failings in risk-based strategies, this has been re-examined, and there are now cost-effectiveness justifications for universal testing in pregnancy [13–16]. Overseas data have shown that ED-based viral hepatitis testing is cost-effective [17–19] but Australian data are currently lacking.

We know that large numbers of patients visit EDs in Australia. In one year, there are almost nine million presentations to ED in Australia [20]. Addressing healthcare issues beyond the precipitant for an ED presentation could be offered in a holistic healthcare system. This method could reach OB patients or other difficult-to-access groups. Routine testing in the ED has been demonstrated to be effective in detecting blood borne infections in several countries [19, 21, 22]. However without a mechanism to ensure that testing is performed in all eligible patients without interfering with ED care or relying on human factors, uptake rates are variable and are as low as 27% in one UK study [21]. The world is searching for solutions to solve this implementation challenge and achieve HCV elimination [23].

In response to the implementation challenges in our local community, particularly among the OB population, SEARCH (Screening Emergency Admissions at Risk of Chronic Hepatitis) was developed as a pilot service (July 2018 to February 2019) for the routine testing for viral hepatitis testing in OB and ATSI patients. Positive patients identified after testing in the ED were linked to care with their primary care provider (PCP) or with specialist services. Detailed methods and clinical results have been published elsewhere [22]. The patient groups targeted in SEARCH were, in our clinical experience, at risk of not being identified with traditional testing services or not linked to appropriate care. This is supported by Australian evidence which shows that ATSI patients are more likely to be diagnosed with HCV but have lower rates of RNA testing or treatment compared to nonindigenous Australians [24]. It is recognised that both ATSI and people from culturally and linguistically diverse backgrounds may be missed in traditional screening approaches and these groups are identified as priority populations for testing and treatment [25].

The SEARCH pilot program of testing involved an opt-out consent after information was provided by brochure and verbally at the time of collection of admission bloods by ED staff. Hepatitis serology was then manually added onto already collected and routinely stored samples. Specimens were robotically retrieved from storage and screened for hepatitis by automated analysers. Results were reviewed by the hepatology project officer and LTC of positive cases was co-ordinated by hepatitis nursing staff. A significant number of patients were identified with HCV, including some new diagnoses and many who were known to have HCV but had been lost to follow-up and/or not previously linked to care or treated.

This cost analysis aimed to accurately describe the actual (not modelled) overall costs of the service pilot. It also aimed to report on costs per patient tested, per HCV Ab-positive patient detected and per RNA-positive patient treated. We modelled the effect that proposed service refinements will have (SEARCH 2.0). Sensitivity analysis to examine the effect variation in HCV Ab prevalence and RNA positivity was performed.

## 2. Methods

### 2.1. Costs of SEARCH 1.0

All direct costs from the time of testing until HCV therapy initiation were calculated. Costs were divided into three groups: (1) screening, (2) LTC of positive (and serologically indeterminate) patients, and (3) work up for treatment of RNA-positive patients. All costs were in Australian dollars ($AUD) with 2018 set as the current time.

In several cases, the exact cost of inputs was known (translation and printing costs for patient information, pathology request forms, etc.) and these were priced based on the actual amount paid. For more complex items involving staff time, a human resource study was conducted. This was assessed by real-time monitoring of staff workflow during the pilot. The average time taken to perform specific tasks was assessed (and also a range of possible times with upper and lower time duration). The cost of each task was then calculated using the appropriate staff salary rate.

The price of HCV Ab testing ($7.39 per test) was calculated based on the total annual cost of the reagents required to perform this test divided by the number of tests performed per year. Testing was performed on pre-existing fully automated equipment. This cost is substantially less than the Australian Medicare Benefits Scheme (MBS) rebate paid for this test. The MBS cost accounts for other related costs of testing (including profit, salaries, equipment costs, depreciation, quality assurance, and laboratory certification) though these are less relevant in a public hospital setting. The MBS cost was included in the sensitivity analysis. The cost of other supplementary laboratory investigations performed as part of DDA treatment workup was calculated using the MBS rebate price. This was because in most cases, the patient had left the hospital by the time these tests were performed, and some tests were performed in primary care via external private laboratories.

Follow-up of costs for viraemic patients included nursing and medical review and further biochemistry. Viraemic patients underwent genotyping (as this was a requirement at the time of DAA prescribing). Patients with aspartate aminotransferase to platelet ratio (APRI) [26] greater than 1.0 underwent transient elastography (TE). This threshold was chosen as it has high sensitivity for detecting advanced fibrosis or cirrhosis and was based on contemporaneous international guidelines [27]. The cost of TE was derived from a previous Australian study [28]. Patients found to have cirrhosis underwent ultrasound screening for hepatocellular carcinoma priced using the MBS cost.

In the situation where a patient could not be contacted, at least three attempts were made to engage them. This was estimated to take an average of 60 minutes of nursing time per patient and this cost has been included in the analysis.

### 2.2. Cost Comparison with SEARCH 2.0 Refinements

**Figure 1:**
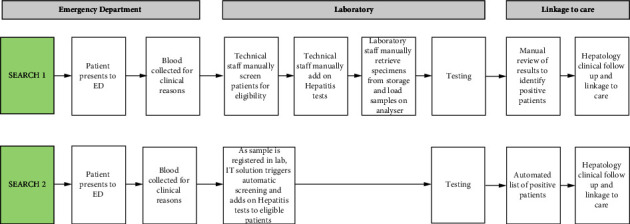
Operational flow of SEARCH 1 compared to SEARCH 2.

Several refinements of the SEARCH pilot (termed SEARCH 1.0) have been designed to improve efficiency and reduce the other direct costs of the HCV Ab screening (Table 1 and Figure 1). These refinements allow the hepatitis testing requests to be linked automatically to the biochemistry order from ED and performed synchronously. This avoids the following steps which were part of SEARCH 1.0; analysis of the demographic of the cohort, selection of OB cases, manual ordering of add on hepatitis test, robotic retrieval and specimen re-run in the analyser. This study will model the effect of these refinements on the cost (using a theoretical model termed SEARCH 2.0).

Following SEARCH 1.0 changes to Australian government requirements for DAA access were made meaning that genotype testing was no longer necessary. As a result, the cost of genotype testing was not included in the SEARCH 2.0 model. This cost saving was not included when estimating the percentage cost reduction achieved through other program efficiencies.

### 2.3. Sensitivity Analysis

In SEARCH 1.0, the HCV Ab prevalence was 3.6%, the rate of RNA positivity was 28% (1% of total tested) and 61% of RNA positive patients were commenced on treatment. For the sensitivity analysis, we modelled HCV Ab prevalence between 0.5 up to 6%, RNA-positive rate of 20 and 40% (of those who were HCV Ab-positive), and treatment commencement rate of between 40% and 70% (of those RNA-positive) (supplementary Table 2).

## 3. Results

**Figure 2:**
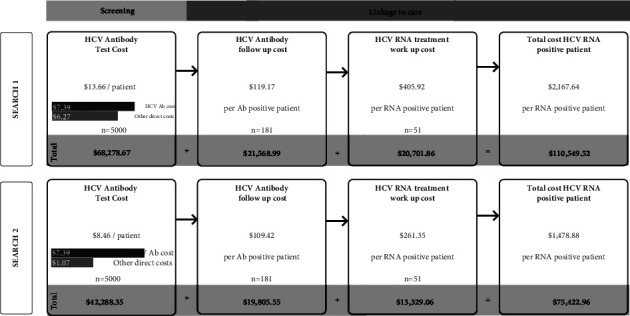
Costs of HCV screening in SEARCH 1 compared to costs modelled with SEARCH 2 program refinements.

The overall cost of the program was calculated to be $110,549.52 which can be broken down into three components: $68,278.67 for the testing of 5000 ED patients, $21,568.99 to follow-up and link HCV Ab-positive patients to care, including the RNA testing, and $20,701.86 to prepare HCV RNA-positive patients for treatment (Figure 2 and Table 2). Per patient at each stage can be expressed as $13.66 per patient screened for HCV Ab which includes the laboratory cost of the test ($7.39 per patient) plus other direct costs ($6.27 per patient), $119.17 per HCV Ab-positive patient followed up and $405.92 per RNA-positive patient worked up for treatment. Overall, the total program cost per HCV Ab-positive patient was $610.77, per HCV RNA-positive patient was $2,167.64, and per patient prescribed DAA therapy was $3,566.11.

### 3.1. Refinements with SEARCH 2.0

Several program refinements have been made to streamline the process following the initial pilot (Figure 1 and Table 1). These allow savings of $35,126.56 in screening 5000 patients. These savings were predominately through internal changes ($27,553.76) of reduced staff time ($27,253.76) and reduced paper costs ($500). External savings of $7,372.80 were also achieved through the elimination of genotype testing as previously described.

Thus, with these refinements in SEARCH 2.0 model, the total program cost to screen 5,000 ED patients was estimated to be $75,422.96 (a 25% cost reduction from SEARCH 1.0 via internal efficiencies). Additionally, by this improved automation, the overall potential cost variability was also reduced (upper and lower estimate: $69,434.48– $82,003.43). Assuming the same community HCV prevalence, RNA positivity and rate of follow-up as found in SEARCH 1.0, the cost of the improved program would be $416.70 per HCV Ab-positive patient, $1,478.88 per RNA-positive patient identified and $2,433.00 per patient prescribed a DAA.

### 3.2. Sensitivity Analysis

#### 3.2.1. Staff Time and Laboratory Costs Sensitivity Analysis

For the majority of costs, the exact price was known (Table 2); however, for items related to staff time a range was considered to provide an estimate of minimum and maximum cost of the program. At the lower end of staff time, the total overall program cost was $92,109.79 and at the upper end was $129,581.24. The exact price of HCV Ab testing was calculated based on laboratory accounts ($7.39 per test); however, the MBS price ($15.65 per test) was also modelled. Using the MBS price would result in a higher overall program cost of $151,849.52.

#### 3.2.2. HCV Antibody Prevalence, RNA-Positive Rate, and Linkage to Care Sensitivity Analysis

**Figure 3:**
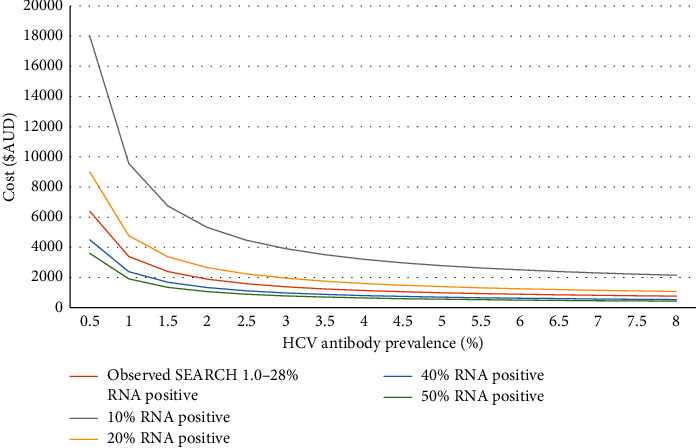
SEARCH 2 estimated cost per RNA-positive patient detected according to HCV antibody prevalence (*x* axis) and the proportion of patients RNA-positive.

Using the SEARCH 2.0 model, the effect on program cost of changes in HCV Ab prevalence, the rate of RNA positivity, and rate of linkage to care were assessed (Figure 3, Table 3, and supplementary Tables 1 and 2). These show all models remain below $10,000 per RNA-positive identified as long as the community prevalence of HCV Ab was greater than 1.0%.

## 4. Discussion

Universal screening is recommended in OB patients from an intermediate or high prevalence country or in patients of ATSI background. However, the implementation of this policy is imperfect, confirmed by recent reports of treatment gaps in these cohorts [4]. Potential barriers to hepatitis testing in these groups include lack of understanding about hepatitis testing recommendations, language barriers, stigma and discrimination, lack of exposure to health promotion campaigns, and reduced health literacy.

Risk-based testing, including a risk based on country of birth, is not well executed. Clinicians may lack knowledge or time (or both) to question patients on risk factors, may not know which countries are classified as high or intermediate risk, may be concerned that patients do not wish to disclose risk factors and consider that risk-based screening does not capture all infected persons [29]. The ED is not a setting where healthcare providers have capacity to perform a blood borne virus risk assessment and engage in guideline-based health promotion and disease prevention activities if not relevant for the presenting problem. Yet, patients do wish and perhaps expect that any visit to a healthcare provider will address significant healthcare needs. Increasingly, the ED is a location where screening for hepatitis is under consideration and evaluation [30].

Although universal screening is not recommended in Australia, the US Preventive Services Task Force (USPSTF) and the Centers for Disease Control recently changed their approach to HCV case finding endorsing universal screening in adults aged 18 to 79 years [31]. Some time ago, analysis of screening and treatment with higher cost, less efficacious drugs required the prevalence of HCV needs to be above 0.84% to be cost-effective [32]; things have changed. In one cost-effectiveness study, universal screening followed by guideline-based treatment of all patients with chronic HCV infection was calculated to cost $11,378 dollars per QALY gained compared to birth cohort screening. In that study, screening costs per QALY gained remained cost-effective as long as the prevalence of HCV Ab was above 0.07% [11]. The incremental cost-effectiveness ratio in fact favoured screening over no testing, meaning universal screening was a cheaper strategy than dealing with the costs of HCV complications resultant for not screening [11, 31]. It has been estimated that 831 patients from the general population need to be screened to save one life [18]. A Markov model of cost-effectiveness of ED testing for hepatitis B or C showed cost-effectiveness, as long as the prevalence was above 0.25% [17]. It should be acknowledged these data are from the USA and results may not be generalisable to Australia.

In our SEARCH pilot, the prevalence of HCV Ab was 3.6%, well above this prevalence threshold for cost-effectiveness. Additionally, as the costs of assessment and treatment are less in Australia than the USA, the cost-effectiveness of universal testing is likely to be even better in Australia.

In this study, we determined the actual cost when we screened all OB and ATSI in the ED. With SEARCH 2.0 refinements, this was found to be $1,479 per RNA-positive patient identified and $2,433 per patient treated. However, how do we decide if this cost is reasonable? The Australian government successfully negotiated a capped price for HCV therapy, which was intended to allow access for all, and achieve HCV elimination with a per-patient treatment course estimated to be approximately $14,000 [33]. The cost of finding patients is not defined and is left to individual clinicians to execute, based on their hepatitis testing approach, guided by recommendations and encouraged by health promotion organisations.

Based on the costs reported here and previous cost-effectiveness models, consideration could be given to applying the SEARCH program more broadly. Within our Local Health District in 2020, there were more than 250,000 individual ED patient presentations [34]. If screening using the SEARCH 2.0 automated strategy was applied to approximately 75% of these presentations or 187,500 patient visits (to exclude paediatric, very elderly and ineligible patients), the costs of the laboratory HCV Ab testing alone would be $1,586,250. The cost per patient found to be RNA-positive would vary depending on the HCV Ab prevalence and the proportion of patients who are RNA-positive (Figure 3), as was explored in the sensitivity analysis.

One limitation of this study is the difficulty in estimating costs of individual items when delivered by existing staff within a health system, when the testing is part of their normal duties and largely automated (such as the HCV Ab test) or low volume (such as the HCV treatment). For SEARCH 2.0, no additional staff need to be recruited to deliver the service and therefore it could be delivered with the cost of reagents being the only factor for consideration.

Our sensitivity analysis has attempted to consider the cost with a range of HCV Ab and RNA prevalences compared to our prevalence of 3.6% and 28%, respectively. We have also modelled the impact of reduced LTC compared to our high rates (over 85% and 61% started DAA). As LTC diminishes, the cost of the program per patient treated escalates and the overall value of the program diminishes. We do not know the actual prevalence of HCV or the likelihood of good LTC in populations other than the OB and ATSI within our hospital. LTC after risk-based screening in an ED in Victoria, Australia was frustratingly low because the patients identified were often homeless, difficult to contact or disengaged from healthcare strategies such as HCV treatment, many without a telephone or address [35]. Efforts to ensure good LTC for marginalised individuals will be an important factor in achieving HCV elimination. Reports of LTC from ED screening in other countries and are more promising [21].

This study examined the utility of a universal testing strategy in OB populations as supported by the current Australian testing policy. Certainly, nontargeted universal testing has been shown to reach many patients who were outside the traditional risk groups [36]. It is likely that testing Australians universally will reveal more cases of hepatitis than risk-based screening. The costs will increase proportionally to the number tested and the utility will depend upon the prevalence in the population. Our analysis suggests this will remain worthwhile even at a lower prevalence than was found in our OB cohort.

In conclusion, the cost of screening patients for HCV Ab in a semiautomated fashion within an ED is low, as little as $7.39 per test or up to $13.66 if other direct costs are included. The other direct costs can be reduced as we modelled in SEARCH 2.0 so that the overall cost per patient screening in the ED is $8.46 per patient tested. The cost of the program expressed as a cost per HCV Ab positive or HCV RNA positive patient found vary depending on the prevalence. The benefit relies on good linkage to care. Modelling has demonstrated that the costs are justifiable, and we have demonstrated before that the program is implementable, if introduced in an automated, opt-out fashion.

## Figures and Tables

**Table 1:** Refinements made between SEARCH 1.0 and SEARCH 2.0 and estimated associated cost savings.

	SEARCH 1.0	SEARCH 2.0	Efficacy	Cost saving
Internal changes				
Test ordering	Manual “adding on” of tests to eligible patients	IT solution to automate test ordering	Technical staff time + paper request form	$5,790.32
HCV antibody testing	Manual retrieval of specimens from storage for testing	Automated testing	Laboratory staff time	$20,200
Reporting	Manual extraction of results	Computer generated list of positive and indeterminate results	Technical staff time	$1,763.44
Total internal cost savings				$27,753.76

External changes				
HCV genotype	Routine for all RNA-positive patients^*∗*^	Not performed	Reduced testing costs	$7,372.80
Total savings				$35,126.56

^*∗*^In 2019, this was a requirement for access to government subsidised direct acting antiviral therapy within Australia. This requirement has subsequently been removed. This saving was not included when calculating the percentage saving achieved through program efficiencies. HCV, hepatitis C virus, RNA, ribonucleic acid.

**Table 2:** Detailed costs of items included in SEARCH 1.0 cost analysis and source of information.

	Item	Cost	Lower bound	Upper bound	Source and explanation
*ED screening*					
	Patient education materials	$1,781.55	—	—	Supplier receipt
	Request form cost^*∗*^	$500	—	—	Supplier receipt ($0.1 per request x5000)
	Manual ordering of tests^*∗*^	$5,290.32	$3,526.88	$7,053.76	90 minutes per day (range 60–120 minutes)
					134 weekdays program ran
					Hourly rate of pay: $26.32
	Laboratory staff manually retrieving samples^*∗*^	$20,200	$10,100	$30,300	6 minutes per sample (range 3–9 minutes) x5000 samples
					Hourly rate of pay: $41.40
	HCV antibody testing	$36,950	—	—	Laboratory cost ($7.39 × 5000).
	HCV supplementary antibody testing	$3,556.80	—	—	MBS 69484 ($17.10 × 208 patients)
					181 positive, 27 indeterminate
Total ED screening costs		**$68,278.67**	**$56,415.23**	**$80,142.11**	

*Follow-up antibody-positive results*					
	Manual review to detect positive test^*∗*^	$1,763.44	$1,175.63	$2,351.25	30 minutes per weekday (range 20–40 minutes)
					134 weekdays program ran
					Hourly rate of pay: $26.32
	Positive patient follow-up (review and explain results, discuss further testing)	$8,872.20	$4,436.10	$13,308.30	1 hour of nursing time (range 30–90 minutes)
					180 patients (1 patient deceased prior to follow-up)
					Hourly rate of pay: $49.29
	Repeat antibody testing of indeterminate cases	$422.55	—	—	MBS 69475 ($15.65 × 27 patients)
	HCV RNA for antibody positive	$10,510.80	—	—	MBS 69499 ($92.20 × 114 patients)
					114 patients without a clear previous treatment history
Total antibody positive follow-up costs		**$21,568.99**	**$16,545.08**	**$26,592.90**	

*Work up of viraemic patients*					
	Linkage to care	$2,513.79	$1,257.15	$3,770.94	Up to 3 phone calls per patient (20 minutes each) (range: 10–30 minutes)
					51 RNA positive patients
					Hourly rate of pay: $49.29
	Supplementation blood tests (FBC, electrolytes and LFTs, HIV and HBV serology)	$2,291.40	-	-	MBS 65070, 66512 and 69387 ($63.65 × 38)
					36 patients appropriate for treatment
	HCV genotype^*∗*^	$7,372.80	—	—	MBS 69491 ($204.80 × 36)
	Fibroscan	$885.60			Based on previous australian study at 800 scans on machine per year(28) ($24.6 × 36 patients)
	Ultrasound	$2,003.40	—	—	MBS 55036 ($111.30 × 20)
					Performed on all cirrhotic patients prior to treatment
	Nursing review for pretreatment assessment	$887.22	$591.48	$1,774.44	30 minutes of nursing time (range 20–60)
					Hourly rate of pay: $49.29
					36 patients assessed for treatment
	Medical review to start therapy	$4,747.65	—	—	MBS 110 ($153.15 × 31)
					31 patients commenced treatment

Total work-up treatment costs		**$20,701.86**	**$19,149.48**	**$22,846.23**	
Total overall cost		**$110,549.52**	**$92,109.79**	**$129,581.24**	

^*∗*^Areas of cost saving in SEARCH 2.0. MBS, medical benefits schedule; HCV, hepatitis C virus; RNA, ribonucleic acid; FBC, full blood count; LFTs, liver function tests; HIV, human immunodeficiency virus; HBV, hepatitis B virus.

**Table 3:** Cost (AUD$) per patient RNA-positive patient detected under several scenarios and variable HCV antibody prevalence.

HCV antibody prevalence (%)	0.5	1	1.5	2.0	2.5	3.0	3.5	4.0	4.5	5.0	5.5	6.0
10% RNA-positive	$18,014	$9,554	$6,734	$5,324	$4,478	$3,914	$3,511	$3,209	$2,974	$2,786	$2,632	$2,504
20% RNA-positive	$9,007	$4,777	$3,367	$2,662	$2,239	$1,957	$1,756	$1,605	$1,487	$1,393	$1,316	$1,252
28% RNA-positive (observed SEARCH 1.0)	$6,393	$3,391	$2,390	$1,890	$1,589	$1,389	$1,246	$1,139	$1,056	$989	$934	$889
40% RNA-positive	$4,504	$2,389	$1,684	$1,331	$1,120	$979	$878	$802	$744	$697	$658	$626
50% RNA-positive	$3,603	$1,911	$1,347	$1,065	$896	$783	$702	$642	$595	$557	$526	$501

^*∗*^Does not include costs of work-up for RNA-positive patients for direct-acting antiviral treatment. AUD, Australian dollars; RNA, ribonucleic acid; HCV, hepatitis C virus.

## Data Availability

The data used to support the findings of this study are available from the corresponding author upon request.

## Supplementary Materials

Supplementary Materials Supplementary Table 1: SEARCH 1.0 cost summary by area and per patient and modelled cost for SEARCH 2.0 for 5000 patients screened. Supplementary Table 2: Cost (AUD$) per patient started on DAA under several scenarios and variable HCV antibody prevalence.
