# Supplementary material for: Finding Cases of Hepatitis C for Treatment Using Automated Screening in the Emergency Department is Effective, but What Is the Cost?
Source: Can J Gastroenterol Hepatol. 2022 Oct 14;2022:3449938. doi: 10.1155/2022/3449938 (PMC9586809; doi:10.1155/2022/3449938)
Supplement: Supplementary Materials — Supplementary Table 1: SEARCH 1.0 cost summary by area and per patient and modelled cost for SEARCH 2.0 for 5000 patients screened. Supplementary Table 2: Cost (AUD$) per patient started on DAA under several scenarios and variable HCV antibody prevalence. [file 3449938.f1.docx]

# Supplementary materials

Supplementary table 1 – SEARCH 1.0 cost summary by area and per patient and modelled cost for SEARCH 2.0 for 5000 patients screened

| Area | Number of patients | SEARCH 1.0 cost | SEARCH 1.0  cost per patient | SEARCH 2.0 modelled cost | SEARCH 2.0 modelled cost per patient |
| --- | --- | --- | --- | --- | --- |
| ED screening | 5000 | $68,278.67 | $13.66 | $42,288.35 | $8.46 |
| Follow up for positive patients | 181 | $21,568.99 | $119.17 | $19,805.55 | $109.42 |
| Work up of RNA positive patients for treatment | 51 | $20,701.86 | $405.92 | $13,329.06 | $261.35 |
| Overall cost | 5000 | $110,549.52 | $22.11 | $75,422.96 | $15.08 |

Abbreviations: ED – emergency department, RNA – Ribonucleic acid

Supplementary table 2 – Cost (AUD$) per patient started on DAA under several scenarios and variable HCV antibody prevalence

| HCV antibody prevalence (%) | 0.5 | 1 | 1.5 | 2.0 | 2.5 | 3.0 | 3.5 | 4.0 | 4.5 | 5.0 | 5.5 | 6.0 |
| --- | --- | --- | --- | --- | --- | --- | --- | --- | --- | --- | --- | --- |
| Observed | 10,948 | 6,008 | 4,362 | 3,539 | 3,045 | 2,715 | 2,480 | 2,304 | 2,166 | 2,057 | 1,967 | 1,892 |
| Scenario A | 1,5424 | 8,465 | 6,145 | 4,985 | 4,289 | 3,825 | 3,494 | 3,245 | 3,052 | 2,897 | 2,771 | 2,666 |
| Scenario B | 7,712 | 4,232 | 3,073 | 2,493 | 2,145 | 1,913 | 1,747 | 1,623 | 1,526 | 1,449 | 1,385 | 1,333 |
| Scenario C | 1,3309 | 7,304 | 5,303 | 4,302 | 3,701 | 3,301 | 3,015 | 2,801 | 2,634 | 2,500 | 2,391 | 2,300 |
| Scenario D | 9,507 | 5,217 | 3,788 | 3,073 | 2,644 | 2,358 | 2,154 | 2,000 | 1,881 | 1,786 | 1,708 | 1,643 |
| Scenario E | 18,537 | 10,077 | 7,257 | 5,847 | 5,001 | 4,437 | 4,034 | 3,732 | 3,497 | 3,309 | 3,155 | 3,027 |

Observed – 28% RNA positive, 61% of RNA positive commenced on DAA, Scenario A – 20% RNA positive and 61% commenced on DAA, Scenario B – 40% RNA positive and 61% commenced on DAA, Scenario C – 28% RNA positive and 50% commenced on DAA, Scenario D – 28% positive and 70% commenced on DAA, Scenario E – 20% RNA positive and 50% commenced on DAA

Abbreviations: AUD – Australian dollars, DAA – direct acting antiviral, HCV – hepatitis C virus, RNA – Ribonucleic acid
